# Supplementary material for: Inhibition of Lipopolysaccharide-Induced Inflammatory Signaling by Soft Coral-Derived Prostaglandin A2 in RAW264.7 Cells
Source: Mar Drugs. 2022 May 9;20(5):316. doi: 10.3390/md20050316 (PMC9143063; doi:10.3390/md20050316)
Supplement: Supplementary file 1 [file marinedrugs-20-00316-s001.zip › marinedrugs-1651399-supplementary.pdf]

marine drugs

Supplementary Materials for

Inhibition of Lipopolysaccharide-Induced Inflammatory Signaling by Soft Coral-Derived Prostaglandin A_2 in RAW264.7 Cells

Osamu Ohno*, Eika Mizuno, Junichiro Miyamoto, Tomoyuki Hoshina, Takuya Sano and Kenji Matsuno*

Department of Chemistry and Life Science, School of Advanced Engineering, Kogakuin University, 2665-1 Nakano, Hachioji, Tokyo 192-0015, Japan

Table of Contents

Spectral data for PGA_2 isolated from the soft coral *Lobophytum* sp. ……………..…………………………………………..….2

Figure S1. ^1H NMR spectrum of PGA_2 isolated from the soft coral *Lobophytum* sp. (400 MHz, $CDCl_3$). …………...………..2

Figure S2. ^1H NMR spectrum of PGA_2 isolated from the soft coral *Lobophytum* sp. (400 MHz, CD_3OD). ……………………3

Figure S3. ^{13}C NMR spectrum of PGA_2 isolated from the soft coral *Lobophytum* sp. (100 MHz, CD_3OD). ……………..…….3

Figure S4. Effects of 10 μM PGA_2 on LPS-induced degradation and resynthesis of IκBα……………………..………………..4

Figure S5. Effects of PMB on LPS-induced degradation of IκBα…………………………………………………………………4

Spectral data for PGA_2 isolated from the soft coral *Lobophytum* sp.

$[\alpha]_D^{25}$ = +1.82 (*c* = 0.34, CH_3OH), EIMS *m*/*z* 334 $[M]^+$, ^1H NMR (400 MHz,$CDCl_3$) δ_H 7.50 (dd, *J* = 5.9, 2.3 Hz, 1 H), 6.18 (dd, *J* = 5.4, 2.3 Hz, 1 H), 5.62 (m, 1 H), 5.62 (m, 1 H), 5.43 (m, 1 H), 5.43 (m, 1 H), 4.13 (ddm, *J* = 12.5, 6.1 Hz, 1 H), 3.26 (m, 1 H), 2.49 (m, 1 H), 2.36 (t, *J* = 7.3 Hz, 2H), 2.33 (m, 1 H), 2.17 (m, 1 H), 2.13 (m, 2 H), 1.71 (tt, *J* = 7.3, 7.3 Hz, 2 H), 1.53 (m, 2 H), 1.30 (m, 2 H), 1.30 (m, 2 H), 1.30 (m, 2 H), 0.89 (t, *J* = 6.8 Hz, 3 H), ^1H NMR (400 MHz,CD_3OD) δ_H 7.65 (dd, *J* = 5.4, 2.3 Hz, 1 H), 6.17 (dd, *J* = 5.4, 1.8 Hz, 1 H), 5.60 (m, 1 H), 5.60 (m, 1 H), 5.44 (m, 1 H), 5.39 (m, 1 H), 4.01 (ddd, *J* = 10.4, 6.3, 1.8 Hz, 1 H), 3.31 (m, 1 H), 2.51 (m, 1 H), 2.29 (t, *J* = 8.6 Hz, 2 H), 2.28 (m, 1H), 2.16 (m, 1 H), 2.13 (m, 2 H), 1.67 (m, 2 H), 1.49 (m, 2 H), 1.32 (m, 2 H), 1.32 (m, 2 H), 1.32 (m, 2 H), 0.91 (t, *J* = 6.8 Hz, 3 H), ^{13}C NMR (100 MHz, CD_3OD) δ_C 213.1, 178.0, 168.2, 136.6, 133.7, 132.3, 131.2, 127.8, 73.2, 53.4, 51.2, 38.3, 34.8, 33.0, 28.2, 27.7, 26.3, 26.2, 23.7, 14.4.

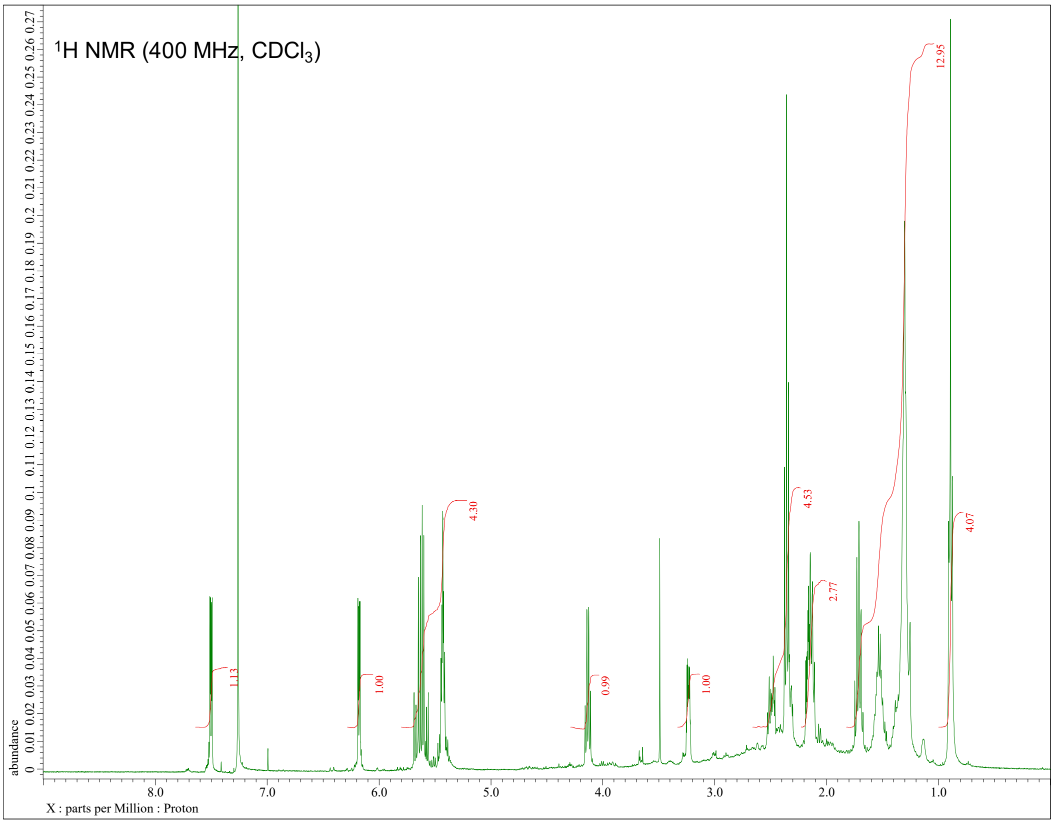

Figure S1. ^1H NMR spectrum of PGA_2 isolated from the soft coral *Lobophytum* sp. (400 MHz, $CDCl_3$).

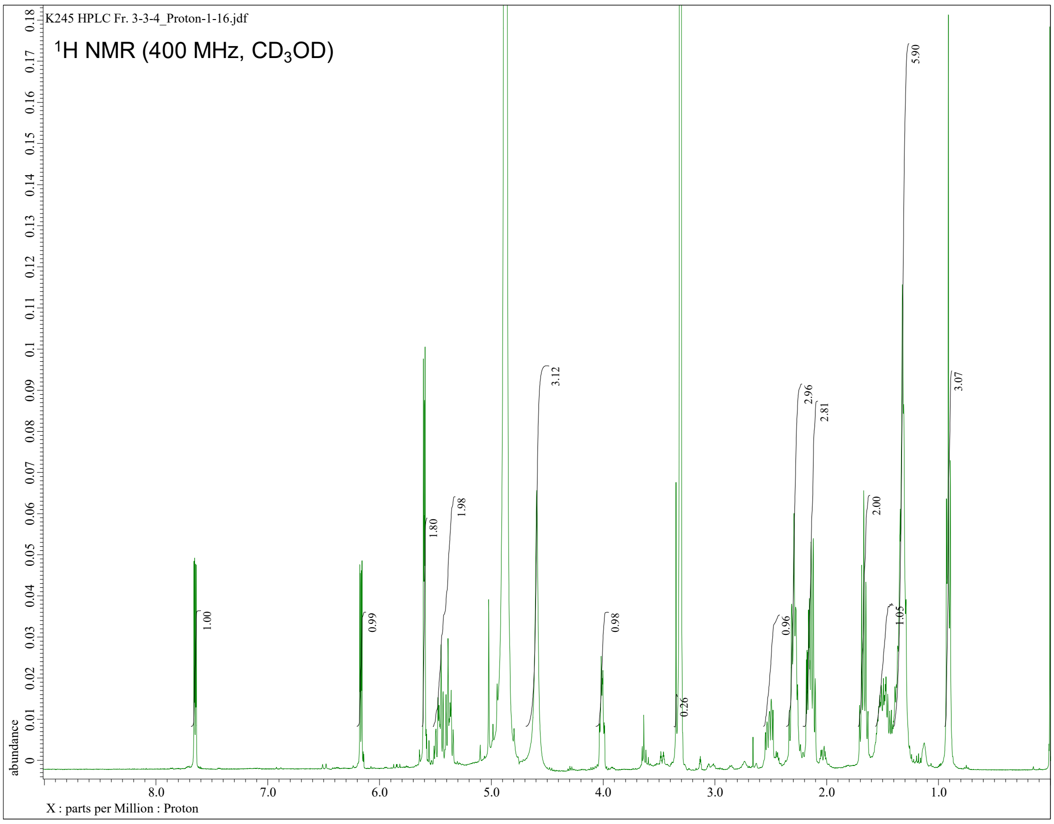

Figure S2. ^{1}H NMR spectrum of PGA_2 isolated from the soft coral *Lobophytum* sp. (400 MHz, CD_3OD).

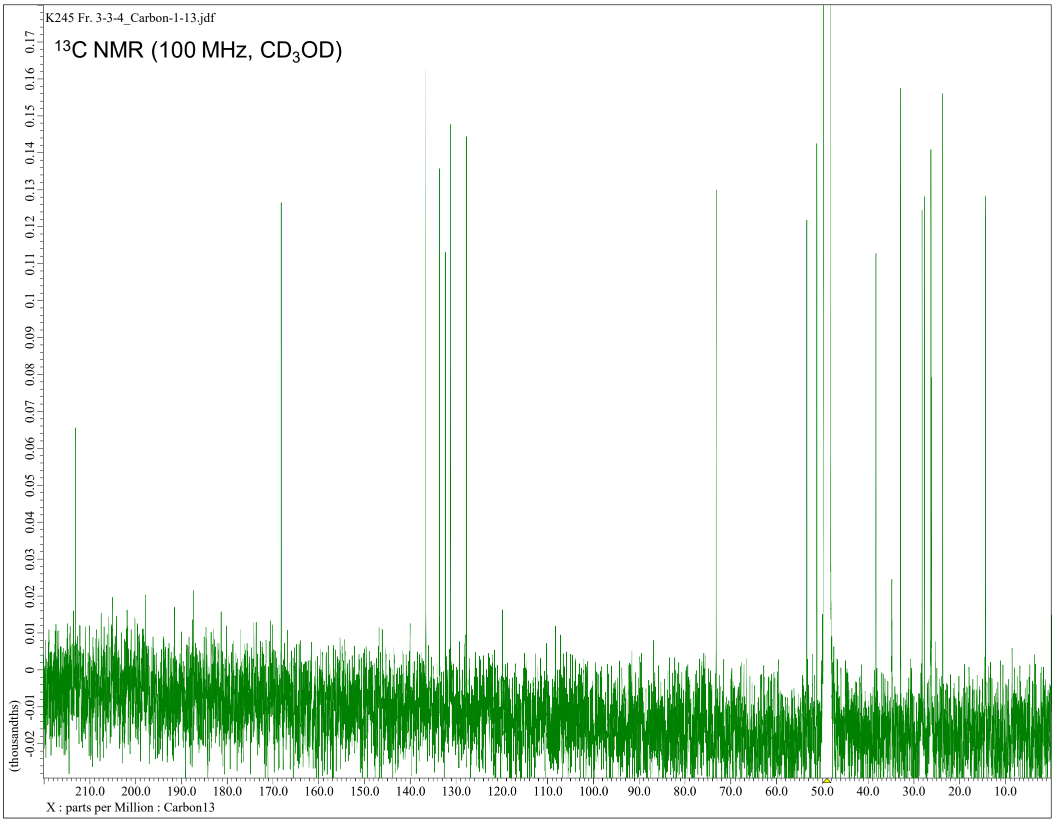

Figure S3. ^{13}C NMR spectrum of PGA_2 isolated from the soft coral *Lobophytum* sp. (100 MHz, CD_3OD).

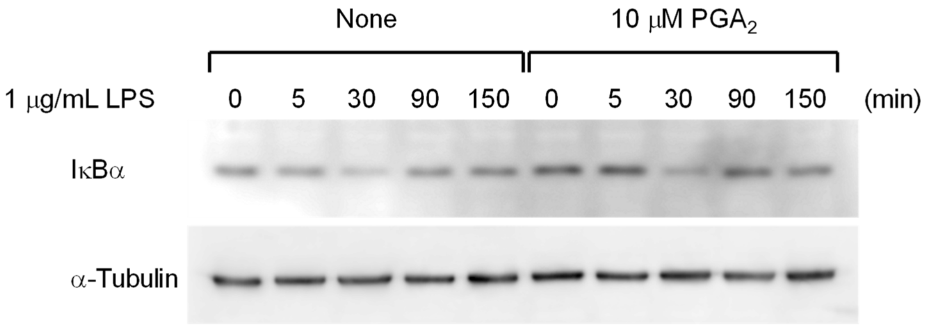

Figure S4. Effects of 10 μM PGA_2 on LPS-induced degradation and resynthesis of IκBα. RAW264.7 cells (1 × 10^6) were preincubated with or without 10 μM PGA_2 for 20 min, then treated with 1 μg/mL LPS for the indicated periods. The cell lysates were analyzed by western blotting with antibodies against IκBα and α-tubulin.

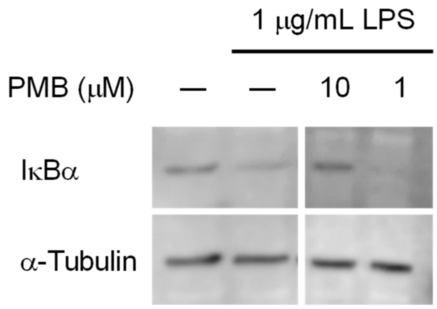

Figure S5. Effects of PMB on LPS-induced degradation of IκBα. RAW264.7 cells (1 × 10^6) were pretreated with the indicated concentrations of PMB at 20 min prior to exposure to 1 μg/mL LPS for 30 min. Then, the cell lysates were analyzed by western blotting with antibodies against IκBα and α-tubulin.
